# Effects of warm water footbath on sleep quality and fatigue in patients with heart failure: a randomized controlled study

**DOI:** 10.3389/fcvm.2026.1834981

**Published:** 2026-09-23

**Authors:** Suh-Meei Hsu, Hsing-Yu Yang, Shu-Yuan Liang, Chieh-Yu Liu, Chung-Lieh Hung, Tsae-Jyy Wang

**Affiliations:** 1 Department of Nursing, MacKay Memorial Hospital, Taipei, Taiwan; 2 Nursing and Management, Mackay Junior College of Medicine, Taipei, Taiwan; 3 School of Nursing, National Taipei University of Nursing and Health Sciences, Taipei, Taiwan; 4 School of Nursing, MacKay Medical University, New Taipei City, Taiwan; 5 College of Nursing, National Yang Ming Chiao Tung University, Taipei, Taiwan; 6 Division of Cardiology, Departments of Internal Medicine, Mackay Memorial Hospital, Taipei, Taiwan; 7 School of Medicine, MacKay Medical University, New Taipei City, Taiwan; 8 Deprtment of Nursing, National Tainan Junior College of Nursing, Tainan City, Taiwan

**Keywords:** actigraphy, fatigue, footbath, heart failure, local heat therapy, sleep quality

## Abstract

**Introduction:**

Poor sleep quality and fatigue are common and clinically important problems in patients with heart failure (HF). Warm water footbaths are a form of local heat therapy that can induce comfort, relieve fatigue, and improve insomnia through heat conduction. This study aimed to explore the effects of a warm-water footbath (WWFB) on sleep quality and fatigue in patients with HF.

**Methods:**

A total of 86 patients with HF were randomized to the WWFB group (*n* = 43) or the control group (*n* = 43), and 74 completed follow-up. The WWFB group received a 42 °C warm-water footbath for 20 min each night for four weeks in addition to actigraphy monitoring, whereas the control group underwent actigraphy monitoring alone. Objective sleep parameters were recorded during the 4-week intervention, and subjective sleep quality and fatigue were assessed at baseline, week 4, and week 8.

**Results:**

Compared with the control group, the WWFB group showed greater improvement in subjective sleep quality and fatigue over time. For objective sleep outcomes, sleep efficiency improved on several study days, and sleep latency was shorter on day 2, whereas total sleep time did not differ significantly between groups.

**Conclusions:**

WWFB is a non-invasive and simple intervention that was associated with improved subjective sleep quality and reduced fatigue in this study. These findings support WWFB as a potential adjunct to home or clinical care for patients with HF.

**Clinical Trial Registration:**

https://clinicaltrials.gov/study/NCT06287996, ClinicalTrials.gov, identifier NCT06287996.

## 1 Introduction

Heart failure (HF) is a complex clinical syndrome defined by structural or functional cardiac impairment that compromises the heart's ability to fill or eject sufficient blood to meet the body's metabolic demands (1, 2). As the final common pathway for most cardiac diseases, HF is a substantial global health and economic burden. The prevalence of HF ranges from 1.5% to 2.0% in developed countries, but increases dramatically to ≥10% among individuals aged ≥70 years (3). In the United States, approximately 6 million adults are affected, with the prevalence projected to increase by 46% by 2030, affecting over 8 million individuals (4). The severity of HF is reflected in its high utilization of healthcare resources; it accounts for over 1 million hospitalizations annually in the United States, with a rehospitalization rate of approximately 25% within the first month after discharge. Consequently, HF is associated with a 5-year mortality rate approaching 48% and total annual costs exceeding 30 billion USD (5).

Beyond its high mortality and economic burden, HF significantly impairs patients' quality of life (QoL), primarily because of the persistent burden of poor sleep quality and fatigue. Sleep disturbances affect nearly 75% of patients with HF and commonly manifest as difficulty initiating or maintaining sleep, early awakening, and daytime dysfunction, often precipitated by symptoms such as dyspnea and nocturia (6, 7). Poor sleep quality is strongly associated with decreased QoL and increased risk of adverse health outcomes (8). Likewise, fatigue is a prevalent and debilitating symptom, reported as moderate to severe by approximately 50% of patients with HF (9). Fatigue reflects inadequate muscle perfusion and serves as an indicator of reduced cardiac output (10). Both poor sleep quality and fatigue are closely interrelated; self-reported poor sleep quality is associated with increased fatigue (11), and fatigue itself is a known predictor of rehospitalization and mortality in this population (12).

The relationship between HF and these symptoms may be related to a cycle of hemodynamic compromise. Reduced cardiac output resulting from cardiac dysfunction can activate the renin–angiotensin–aldosterone system (RAAS) and the sympathetic nervous system (SNS). Increased SNS activity may stimulate the brain's arousal system, thereby contributing to sleep disturbance, whereas tissue hypoperfusion and prolonged SNS activation may contribute to fatigue (13, 14). To manage these symptoms, various non-pharmacological strategies have been explored, including exercise training (such as inspiratory muscle training and Baduanjin exercise), cognitive behavioral therapy for insomnia (CBT-I), and massage therapy (15–17). However, complex interventions, such as CBT-I and unsupervised exercise programs, often face challenges related to patient adherence and long-term sustainability.

Given the limitations of complex treatment regimens, there is a need for easily implementable low-cost self-care strategies. Warm water foot baths (WWFBs), a non-invasive relaxation therapy involving localized heating of the lower extremities, represent a highly feasible alternative. WWFBs have been hypothesized to improve sleep through peripheral temperature regulation and relaxation. Increased peripheral temperature may influence thermoregulation and promote sleep initiation (18). WWFBs may also alleviate fatigue by modulating autonomic nervous system activity, including decreased sympathetic activity and increased parasympathetic tone, which may contribute to vasodilation and improved tissue perfusion (18, 19). Despite these plausible mechanisms, the existing literature reveals a critical gap; no studies have specifically investigated the effects of WWFBs on both sleep quality and fatigue in patients with HF. Therefore, this study aimed to evaluate the effects of WWFBs on sleep quality and fatigue in patients with HF, thereby providing evidence for a simple, effective adjunctive therapy applicable in both hospital-based and home self-care settings to improve patient QoL.

## 2 Methods

### 2.1 Study trial

This study employed a randomized controlled trial design. Patients with chronic HF (CHF) were randomized in a 1:1 ratio to either a WWFB group or a control group. The intervention lasted four weeks, and outcomes were assessed at baseline, week 4, and week 8. The primary objective of the trial was to determine whether WWFB could improve objective sleep quality (total sleep time, sleep latency, and sleep efficiency), subjective sleep quality, and fatigue in patients with CHF.

The trial was approved by the Institutional Review Board of MacKay Memorial Hospital, Taiwan (Approval Number: 23MMHIS503e; date of approval: 06 March 2024), and was registered at ClinicalTrials.gov (NCT06287996). All participants volunteered after receiving oral and written information and provided written informed consent in accordance with the Declaration of Helsinki.

### 2.2 Participants

A total of 86 participants, both sexes, were recruited from cardiology outpatient clinics at two medical centers in northern Taiwan. Patients with CHF were eligible if they met the following criteria: (i) age ≥ 18 years; (ii) New York Heart Association (NYHA) functional class I–III; (iii) consciousness and ability to communicate; (iv) ability to maintain a sitting posture independently or with assistance; and (v) normal body temperature, hearing, and vision. Exclusion criteria were: (i) leg injury or foot wounds; (ii) peripheral vascular disease or reversible CHF; and (iii) history of stroke.

### 2.3 Intervention

#### 2.3.1 Warm water foot bath group

The WWFB group received a four-week WWFB intervention. In addition to routine care and wearing an actigraphy device, participants soaked their feet in a foot bath nightly, approximately 1 h before bedtime (between 7:00 and 9:00 pm). Throughout the study period, the same electronic thermometer was used to confirm the water temperature before each session to ensure intervention consistency and prevent burns. Participants and their primary caregivers received standardized education and instruction regarding the intervention procedure using a “Warm Water Foot Bath Intervention Education Sheet.” Each participant was provided with a foldable intelligent constant-temperature foot bath, an electronic thermometer, and an implementation record sheet. Participants were instructed to prepare the foot bath water at 42 °C at home and confirm the temperature using the thermometer before each session. No soap, salt, herbal products, essential oils, or other additives were added to the water during the intervention. Participants were instructed to inspect their feet for swelling, wounds, broken skin, or other discomfort before each session. After confirming the water temperature, participants were instructed to sit quietly in a comfortable seated position and immerse both feet in the foot bath for 20 min, with the water level maintained approximately 10 cm above the ankle. Participants were instructed to avoid engaging in other activities during the intervention, which was performed at home under unsupervised conditions. To improve intervention consistency, participants were advised to perform the footbath in a quiet and comfortable environment before bedtime. Adherence was monitored using participant-completed implementation record sheets and weekly telephone follow-up interviews conducted by the same researcher throughout the study period. If any discomfort occurred during the foot bath (e.g., dizziness, nausea, chest tightness, dyspnea, or discomfort related to heat exposure), then the procedure was stopped immediately, and participants were instructed to rest and monitor their physical condition.

#### 2.3.2 Control group

The control group received routine care and wore an actigraphy device for four weeks. Routine care included standard outpatient HF management, medication treatment, and routine nursing education provided by the cardiology clinics. Participants and their primary caregivers received an “Actigraphy Health Education Sheet.” Participants in the control group did not receive WWFB intervention instructions or sleep hygiene education. They were asked not to perform additional footbath therapy during the study. Weekly telephone follow-up was conducted to monitor adherence and minimize contamination between groups.

### 2.4 Outcomes

The primary outcome of the trial was sleep quality during the intervention period. Secondary outcomes included changes in fatigue from baseline to week 8.

#### 2.4.1 Sleep quality

##### 2.4.1.1 Objective sleep quality

Objective sleep quality (total sleep time, sleep latency, and sleep efficiency) was assessed using actigraphy, a device that continuously monitors activity levels. Worn on the non-dominant wrist, the device estimates sleep patterns based on limb movement. This study used the AMOR H2 Plus actigraphy device (Leadtek Research Inc.) licensed by the Ministry of Health and Welfare. The actigraphy device was fitted with researcher assistance at baseline and removed after four weeks. Using proprietary software, the device recorded sleep parameters, including total sleep time, total awake time, number of awakenings, average awakening time, sleep latency, and sleep efficiency. Prior to the study, the researcher received training on device operation from a Taiwanese distributor, and technical support was maintained throughout the study.

##### 2.4.1.2 Subjective sleep quality

Subjective sleep quality was assessed using the Pittsburgh Sleep Quality Index (PSQI) developed by BuysseKupfer (20). This self-administered questionnaire evaluates sleep quality over the previous month and consists of nine items, with item five containing ten sub-items. The PSQI includes seven components: subjective sleep quality, sleep latency, total sleep time, sleep efficiency, sleep disturbances, use of sleeping medication, and daytime dysfunction. Each component was scored from 0 (no difficulty) to 3 (very difficult), yielding a total score ranging from 0 to 21. A total score of >5 indicates poor sleep quality, with higher scores reflecting worse sleep quality. The PSQI demonstrates good reliability and validity, with a Cronbach's *α* of 0.83 and test–retest reliability of 0.85 (21). This study used the Chinese version of the PSQI by Tsai et al. (21). The scale has a content validity index of 0.94, Cronbach's *α* ranging from 0.82 to 0.83, and test–retest reliability of 0.85. With a cutoff score of 5, the sensitivity and specificity were 98% and 55%, respectively.

#### 2.4.2 Fatigue

Fatigue was assessed using the Piper Fatigue Scale, which consists of 22 items across four subscales: behavioral/severity (six items), affective meaning (five items), sensory (five items), and cognitive/mood (six items). In addition, the scale included one open-ended question regarding the duration of fatigue and four open-ended questions addressing factors contributing to fatigue, methods for alleviating fatigue, and related symptoms. The original scale is scored on a 10-point scale, yielding a total score ranging from 22 to 220 (22). Chen et al. (23) adapted and validated a modified Chinese version of the scale for patients with HF. In this version, the scale comprises 15 items rated on a 0–3 scale, with a total score ranging from 0 to 45; higher scores indicate more severe fatigue. The modified scale demonstrated good internal consistency (Cronbach's *α* = 0.85). Accordingly, this study adopted the modified Piper Fatigue Scale, as modified by Chen et al. (23).

### 2.5 Warm water foot bath

A foldable intelligent constant-temperature foot bath (model JKQ-550; dimensions 40 cm × 34 cm × 22 cm) manufactured in China was used. The water temperature was maintained at 42 ℃. The device holds BCTC international certification and is licensed by the Taiwan Bureau of Standards, Metrology, and Inspection. The water depth was maintained at 10 cm above the ankle, and the foot bath duration was 20 min.

### 2.6 Water temperature measuring instrument

To ensure participant safety, a water temperature thermometer was used to confirm the bath temperature. A Japanese-made Dretec water thermometer (model O-238 N), certified by the Japanese inspection authority, was used to provide double verification of the water temperature.

### 2.7 Sample size

Based on a two-sided *α* of 0.05, power of 0.8, and an effect size of 0.25 (f), the required sample size was calculated as 68 participants. The effect size was based on the mean differences in sleep outcomes between the WWFB and control groups reported in previous studies. To account for an anticipated dropout rate of 25%, 86 participants (43 per group) were recruited.

### 2.8 Randomization

Participants were randomly allocated to either the WWFB group or the control group using block randomization with a block size of four and an allocation ratio of 1:1. Computer-generated random numbers were prepared by an independent individual who was not involved in the study. The allocation sequences were sealed in opaque envelopes. A research assistant who collected the baseline data and was blinded to the group allocation opened the envelopes to assign participants to groups. Given the nature of the intervention, participant blinding was not feasible. Statistical analyses were conducted using coded group data to reduce potential bias.

### 2.9 Statistical analysis

Continuous variables are presented as means and standard deviations (SDs) and were compared using independent-sample t-tests. Categorical variables are presented as percentages and were compared using the chi-square test. Changes in sleep and fatigue scores during follow-up were calculated as the difference between follow-up time points (T1 and T2) and baseline (T0). Changes in sleep quality and fatigue over time were analyzed using generalized estimating equation (GEE) models with an intercept. The models included time point, group assignment, and a group-by-time interaction term. GEE models were used to account for dropouts and missing data by incorporating all available repeated-measures data from T0 to T2 for each participant. This approach allowed the inclusion of participants with incomplete follow-up data and partially addressed intention-to-treat principles. GEE analysis accounts for within-subject correlations and missing observations without excluding participants with partial data.

## 3 Results

A total of 205 patients were assessed for eligibility between March and July 2024. Of these, 86 participants were randomized and included in the baseline analyses. Figure 1 illustrates participant recruitment, randomization, dropout, and follow-up at the two time points. The baseline characteristics were similar between the two groups (Table 1). The mean age of the total sample was 60.91 years (SD = 11.92), and 81.10% were male. Most participants (85.1%) reported not using sleeping pills, and 89.2% had chronic diseases. The mean cardiac ejection fraction was 48.20% (SD = 11.83), ranging from 28.10% to 72.90%. The distribution of NYHA functional classes was as follows: Class II, 79.73%, Class I, 10.81%, and Class III, 9.46%.

**Figure 1:**
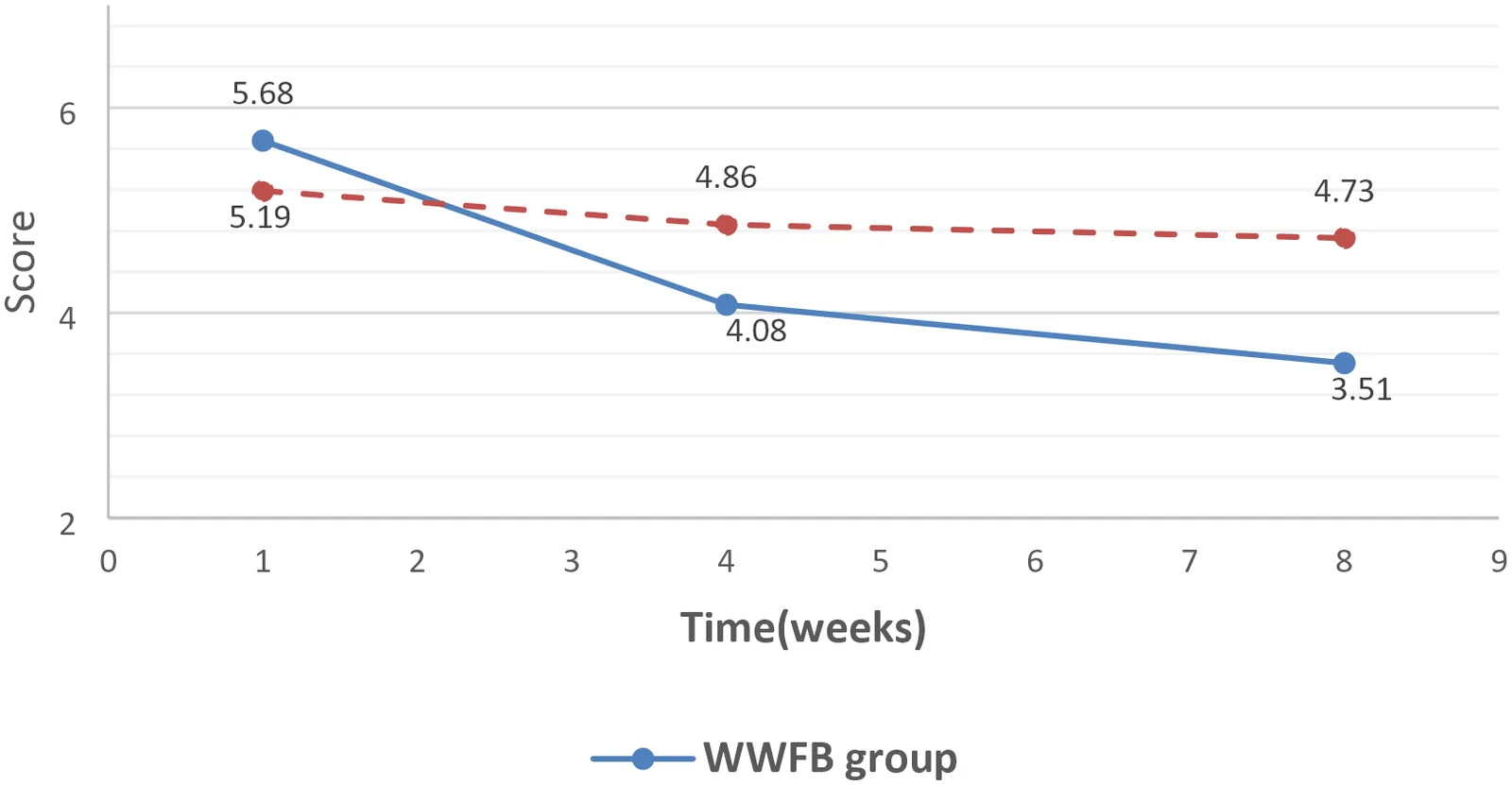
Flowchart of the study participants. First follow-up: after the end of the intervention period (4 weeks); Second follow-up: 8 weeks post-intervention.

**Table 1:** Participant characteristics and demographics at baseline.

Characteristics	Total (*n* = 74)	WWFB group (*n* = 37)	Control group (*n* = 37)	*χ*^2^/t	*p*
	*n* (%)	*n* (%)	*n* (%)		
Age [Mean (SD)]	60.91 (11.92)	59.68 (11.84)	62.14 (12.04)	0.88	0.379
Sex				1.41	0.235
Female	14 (18.90)	9 (24.21)	5 (13.52)		
Male	60 (81.10)	28 (75.80)	32 (86.51)		
Education				1.94	0.746
Primary school	16 (21.70)	10 (27.03)	6 (16.22)		
High	23 (31.10)	11 (29.73)	12 (32.43)		
Junior college/university	24 (32.40)	10 (27.03)	14 (37.84)		
University graduate	11 (14.90)	6 (16.22)	5 (13.51)		
Employment				0.23	0.891
No (retired)	34 (45.90)	16 (43.24)	18 (48.65)		
Full-time	27 (36.50)	14 (37.84)	13 (35.14)		
Part-time	13 (17.60)	7 (18.92)	6 (16.22)		
Marital Status				3.14	0.370
Unmarried	14 (18.90)	5 (13.51)	9 (24.32)		
Married	48 (64.90)	24 (64.86)	24 (64.86)		
Divorced	8 (10.80)	6 (16.22)	2 (5.41)		
Widowed	4 (5.40)	2 (5.41)	2 (5.41)		
Sleep medication				0.107	0.744
Yes	11 (14.90)	6 (16.22) 31 (83.78)	5 (13.51) 32 (86.49)		
No	63 (85.1)				
Medical history				0.00	1.000
Yes	66 (89.20)	33 (81.19)	33 (81.19)		
No	8 (10.80)	4 (10.81)	4 (10.81)		
LVEF (%) [Mean (SD)]	48.40 (11.83)	49.22 (12.88)	47.57 (10.79)	−0.59	0.553
NYHA class					
Class I	8 (10.81)	3 (8.11)	5 (13.51)		
Class II	59 (79.73)	30 (81.08)	29 (78.38)		
Class III	7 (9.46)	4 (10.81)	3 (8.11)		
Class IV	0 (0.00)	0 (0.00)	0 (0.00)		

The demographic and clinical characteristics presented in Table 1 were generally similar between the two groups.

### 3.1 Sleep quality

#### 3.1.1 Objective sleep quality

The AMOR H2 Plus activity tracker was used to continuously monitor the participants' limb activity to estimate sleep status and record sleep parameters, including total sleep time, sleep latency, and sleep efficiency. Each participant wore the tracker for four weeks (days 1–28), generating 28 daily measurements for each sleep parameter (Figure 2). GEE analysis was conducted to examine the differences in total sleep time, sleep latency, and sleep efficiency between the two groups over time. The results showed no significant group-time interaction for total sleep time (Table 2). For sleep latency, GEE analysis revealed no significant group–time interaction, except on day 2. On comparing day 2 with day 1 (T2 vs. T1), the experimental group demonstrated a sleep latency that was 8.952 min shorter than that of the control group, representing a statistically significant difference (*p* = 0.007). Regarding sleep efficiency, significant group–time interactions were observed on nine days (days 2, 9, 11, 12, 13, 18, 23, 24, and 25). Compared with day 1, sleep efficiency in the experimental group increased by 1.403% (*p* = 0.029), 4.468% (*p* = 0.022), 4.428% (*p* = 0.015), 5.375% (*p* = 0.004), 5.605% (*p* = 0.005), 5.114% (*p* = 0.010), 3.519% (*p* = 0.046), 4.794% (*p* = 0.011), and 5.572% (*p* = 0.002), respectively, compared with the control group. These findings suggest that WWFBs were associated with improved sleep efficiency on several study days and shorter sleep latency on day 2, whereas no significant effect was observed on total sleep time.

**Figure 2:**
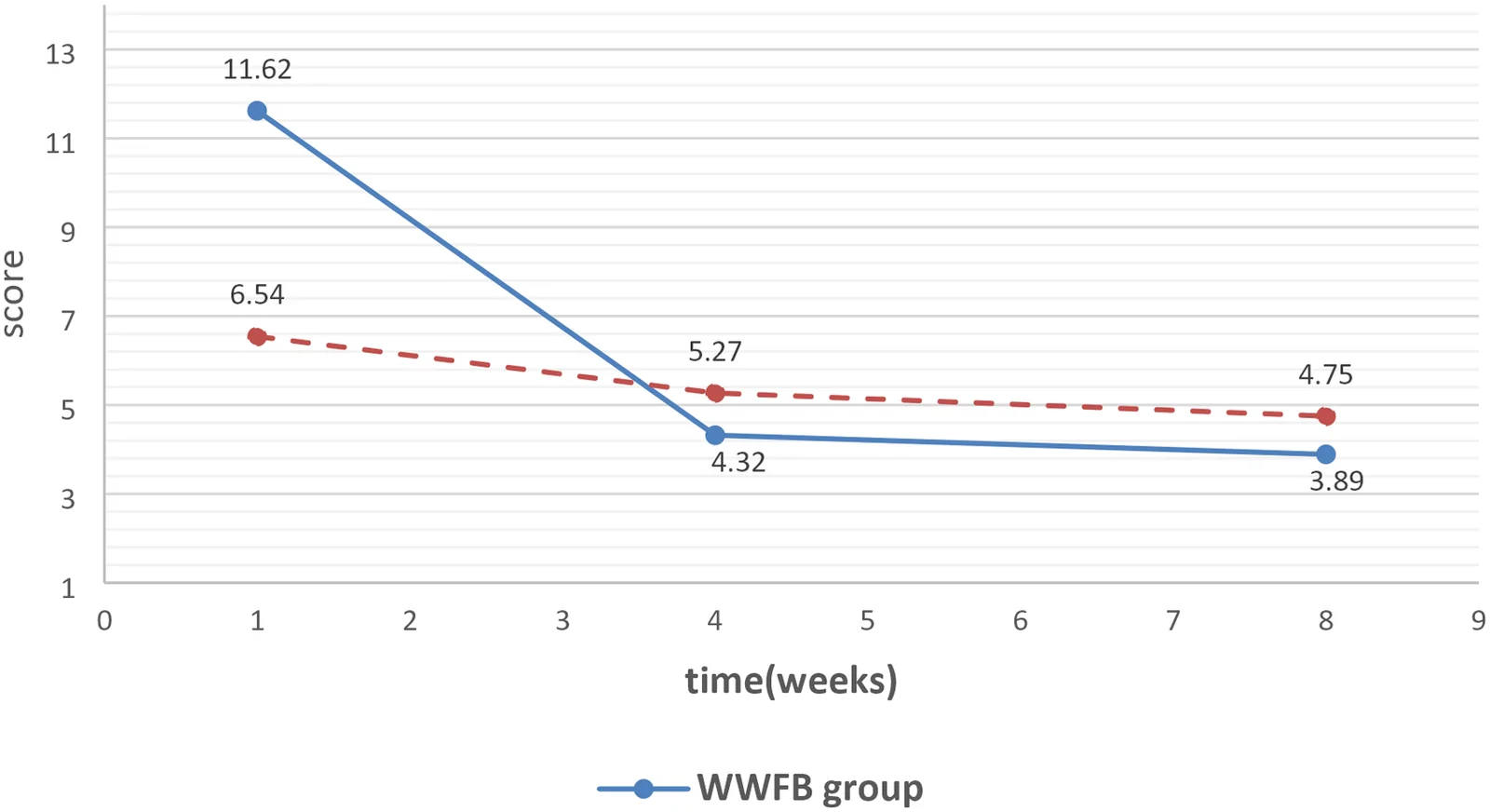
Trend in sleep quality parameters (total sleep time, sleep latency, and sleep efficiency) over four weeks.

**Table 2:** Intergroup comparison of changes in objective sleep quality over time among study participants (*n* = 74).

Parameter	Total sleep time			Sleep latency			Sleep efficiency		
	*B*	*S.E.*	*p*	*B*	*S.E.*	*P*	*B*	*S.E.*	*p*
Intercept	348.27	15.21	0.000	77.06	11.03	0.000	85.75	1.44	0.000
(Exp. vs. Con.)	48.36	25.14	0.054	−41.35	11.88	0.001***	5.89	1.84	0.001***
Time									
(T28 vs. T1)	17.26	14.19	0.224	−2.14	10.98	0.845	−1.11	1.61	0.492
(T27 vs. T1)	6.67	14.09	0.636	3.17	11.17	0.777	−1.88	1.61	0.244
(T26 vs. T1)	0.21	12.65	0.987	2.24	9.99	0.822	−1.82	1.33	0.174
(T25 vs. T1)	9.97	14.13	0.481	4.26	10.34	0.681	−1.88	1.43	0.191
(T24 vs. T1)	1.44	16.58	0.931	1.62	10.61	0.879	−2.07	1.54	0.181
(T23 vs. T1)	8.67	16.52	0.600	−9.70	9.35	0.300	−0.12	1.41	0.934
(T22 vs. T1)	3.85	14.22	0.787	−13.65	9.58	0.154	0.71	1.42	0.620
(T21 vs. T1)	10.12	11.16	0.365	−11.17	9.82	0.255	0.14	1.52	0.928
(T20 vs. T1)	4.73	12.08	0.696	−5.76	9.03	0.523	−0.33	1.42	0.815
(T19 vs. T1)	13.19	14.14	0.351	−5.38	10.19	0.598	−0.74	1.81	0.682
(T18 vs. T1)	0.53	17.07	0.975	−0.36	9.26	0.969	−1.43	1.50	0.343
(T17 vs. T1)	14.55	13.65	0.287	−6.51	9.21	0.480	−0.31	1.47	0.834
(T16 vs. T1)	23.33	12.13	0.055	−10.34	10.40	0.321	0.58	1.48	0.696
(T15 vs. T1)	28.22	13.62	0.038	−7.92	11.53	0.492	0.05	1.81	0.977
(T14 vs. T1)	16.36	13.80	0.236	−9.62	11.31	0.395	0.27	1.77	0.880
(T13 vs. T1)	15.88	13.06	0.224	2.20	11.33	0.846	−2.56	1.73	0.141
(T12 vs. T1)	−7.79	19.03	0.682	4.18	11.69	0.721	−3.27	1.65	0.048
(T11 vs. T1)	6.22	14.74	0.673	6.60	11.49	0.566	−3.67	1.64	0.026
(T10 vs. T1)	12.41	13.86	0.371	1.84	12.35	0.882	−3.21	1.89	0.091
(T9 vs. T1)	27.98	10.60	0.008	3.21	12.40	0.796	−2.99	1.81	0.099
(T8 vs. T1)	0.12	13.46	0.993	−6.34	12.53	0.613	−1.70	1.81	0.349
(T7 vs. T1)	12.96	14.23	0.363	−8.34	11.90	0.484	−0.44	1.81	0.810
(T6 vs. T1)	20.09	13.09	0.125	−8.15	10.08	0.419	0.32	1.40	0.823
(T5 vs. T1)	9.30	12.60	0.460	−12.36	9.23	0.181	1.31	1.17	0.264
(T4 vs. T1)	16.03	11.54	0.165	−14.84	8.77	0.091	1.19	1.1	0.308
(T3 vs. T1)	26.52	12.58	0.035	−4.41	6.81	0.518	−0.37	0.63	0.557
(T2 vs. T1)	25.09	11.47	0.029	6.42	3.07	0.037	−0.69	0.52	0.188
Interaction									
(E vs. C) × (T28 vs. T1)	−52.75	23.47	0.462	−1.21	11.97	0.919	2.33	2.04	0.255
(E vs. C) × (T27 vs. T1)	−49.13	21.21	0.304	−7.42	12.07	0.539	3.18	2.05	0.121
(E vs. C) × (T26 vs. T1)	−44.36	22.86	0.052	−8.28	10.96	0.450	3.52	1.84	0.056
(E vs. C) × (T25 vs. T1)	45.97	62.23	0.460	−17.62	11.04	0.111	5.57	1.76	0.002**
(E vs. C) × (T24 vs. T1)	−13.90	23.12	0.55	−9.350	11.45	0.41	4.794	1.89	0.011*
(E vs. C) × (T23 vs. T1)	−25.31	24.19	0.30	−1.278	10.12	0.90	3.519	1.76	0.046*
(E vs. C) × (T22 vs. T1)	−22.39	23.01	0.33	4.870	10.42	0.64	2.368	1.77	0.182
(E vs. C) × (T21 vs. T1)	−29.35	21.69	0.18	1.117	10.69	0.92	3.013	1.89	0.112
(E vs. C) × (T20 vs. T1)	12.09	22.27	0.59	2.064	10.63	0.85	2.310	1.98	0.245
(E vs. C) × (T19 vs. T1)	−32.65	24.43	0.18	0.886	11.43	0.94	2.562	2.12	0.228
(E vs. C) × (T18 vs. T1)	13.92	29.78	0.64	−12.392	10.52	0.24	5.114	1.97	0.010*
(E vs. C) × (T17 vs. T1)	−40.19	26.87	0.14	−5.413	10.33	0.60	3.757	1.92	0.051
(E vs. C) × (T16 vs. T1)	−55.87	24.82	0.23	−0.734	11.17	0.95	2.644	1.85	0.155
(E vs. C) × (T15 vs. T1)	−27.05	22.63	0.23	−2.283	12.13	0.85	2.576	2.02	0.203
(E vs. C) × (T14 vs. T1)	−2.97	23.23	0.90	0.618	11.84	0.96	2.590	1.99	0.194
(E vs. C) × (T13 vs. T1)	−13.12	22.03	0.55	−10.486	11.86	0.38	5.605	1.98	0.005**
(E vs. C) × (T12 vs. T1)	18.53	28.27	0.51	−7.937	12.26	0.52	5.375	1.88	0.004**
(E vs. C) × (T11 vs. T1)	5.34	24.86	0.83	−6.672	12.13	0.58	4.428	1.81	0.015*
(E vs. C) × (T10 vs. T1)	−18.95	23.87	0.43	−2.904	13.05	0.82	4.102	2.10	0.051
(E vs. C) × (T9 vs. T1)	−35.43	19.54	0.07	−7.628	12.90	0.56	4.468	1.95	0.022*
(E vs. C) × (T8 vs. T1)	8.49	23.62	0.72	2.293	13.02	0.86	2.817	1.99	0.157
(E vs. C) × (T7 vs. T1)	−24.23	21.87	0.27	3.840	12.25	0.75	1.533	1.95	0.434
(E vs. C) × (T6 vs. T1)	−7.02	24.14	0.77	3.297	10.57	0.76	1.235	1.63	0.449
(E vs. C) × (T5 vs. T1)	−14.71	22.01	0.50	7.974	9.68	0.41	−0.127	1.36	0.926
(E vs. C) × (T4 vs. T1)	−27.70	20.81	0.18	10.029	9.20	0.28	0.238	1.36	0.862
(E vs. C) × (T3 vs. T1)	−12.70	21.93	0.56	−0.867	7.16	0.90	1.405	0.77	0.069
(E vs. C) × (T2 vs. T1)	−17.41	19.22	0.37	−8.952	3.33	0.01**	1.403	0.64	0.029*

E., WWFB group; C., Control group; T1, Day 1, T2: Day 2…. T28: Day 28…; GEE, Generalized Estimating Equations for repeated measurements and an AR (1) correlation structure.

* *p* < 0.05.

** *p* < 0.01.

*** *p* < 0.001.

#### 3.1.2 Subjective sleep quality

Lower scores on the PSQI indicate better sleep quality. Both groups completed the PSQI at baseline (T0), week 4 (T1), and week 8 (T2). At baseline, the experimental group had a mean PSQI score of 5.60 (SD = 2.75), while the control group had a mean score of 5.19 (SD = 2.57). An independent-samples t-test analysis showed no significant difference between the groups (*t* = 0.79, *p* = 0.43). At week 4, the mean PSQI score was 4.08 (SD = 2.39) in the experimental group and 4.86 (SD = 2.64) in the control group, with no significant between-group difference (*t* = −1.34, *p* = 0.19). At week 8, the experimental group had a significantly lower mean PSQI score (3.51, SD = 1.90) than the control group (4.73, SD = 2.63), indicating better sleep quality (*t* = −2.28, *p* = 0.03) (Figure 3; Table 3). Further analysis using GEE showed a statistically significant group–time interaction. Compared with the control group, the experimental group demonstrated a significantly greater reduction in PSQI scores from baseline to week 4 (*p* < 0.001) and from baseline to week 8 (*p* < 0.001) (Table 3). These results support the effectiveness of WWFBs in improving subjective sleep quality in patients with HF.

**Figure 3:**
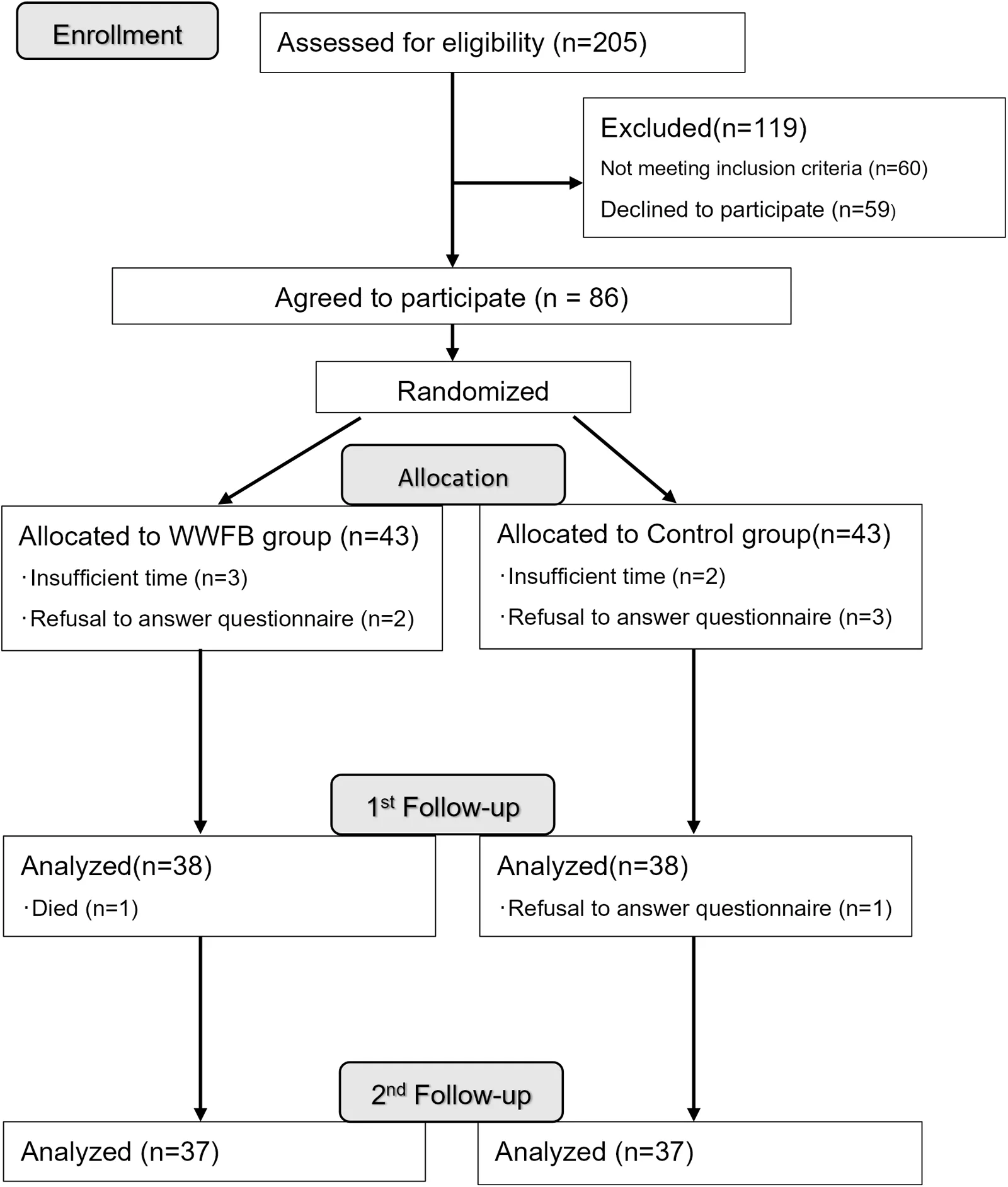
Changes in Pittsburgh sleep quality scale scores among patients with heart failure at pre-test, 4 weeks, and 8 weeks.

**Table 3:** Generalized estimating equation analysis of intervention effects on sleep and fatigue.

Outcome measure	Time	WWFB group (*n* = 37) Mean (SD)	Control group (*n* = 37) Mean (SD)	Group effect		Group * time effect				
				*t*	*p*	*B*	*SE*	95%CI	*χ* ^2^	*p*
Sleep	T0	5.68 (2.75)	5.19 (2.57)	0.79	0.43					
Quality	T1	4.08 (2.39)	4.86 (2.64)	−1.34	0.19	−1.27	0.36	−1.97 to −0.57	12.77	<0.001***
	T2	3.51 (1.90)	4.73 (2.63)	−2.28	0.03*	−1.70	0.39	−2.47 to −0.93	18.70	<0.001***
Fatigue	T0	11.62 (9.31)	6.54 (5.90)	2.80	0.01**					
	T1	4.32 (6.79)	5.27 (5.90)	−0.64	0.53	−6.02	1.39	−8.44 to −3.29	18.60	<0.001***
	T2	3.89 ± 5.06	4.75 ± 5.36	−0.82	0.42	−5.98	1.35	−8.64 to −3.32	19.52	<0.001***

*β*, unstandardized regression *β*-coefficient; 95 CI%, Confidence interval; T1, immediately after intervention (4 weeks); T2, 8 weeks follow-up data collection.

* *p* < 0.05.

** *p* < 0.01.

*** *p* < 0.001.

### 3.2 Fatigue

Both groups completed the Piper Fatigue Scale at baseline, week 4, and week 8. Higher scores indicate more severe fatigue. At baseline, the mean fatigue score was significantly higher in the experimental group (11.62, SD = 9.31) than in the control group (6.54, SD = 5.90) (*t* = 2.80, *p* = 0.01). At week 4, the mean fatigue score was 4.32 (SD = 6.79) in the experimental group and 5.27 (SD = 5.90) in the control group, with no significant difference (*t* = −0.64, *p* = 0.53). At week 8, the mean fatigue score was 3.89 (SD = 5.06) in the experimental group and 4.75 (SD = 5.36) in the control group, with no significant difference (*t* = −0.82, *p* = 0.42) (Figure 4; Table 3). GEE analysis revealed a statistically significant group–time interaction. Compared with baseline, the experimental group showed a 6.02-point greater reduction in fatigue at week 4 than the control group (*p* < 0.001) and a 5.98-point greater reduction at week 8 (*p* = 0.001) (Table 3). These findings support the effectiveness of WWFBs in reducing fatigue in patients with HF.

**Figure 4:**
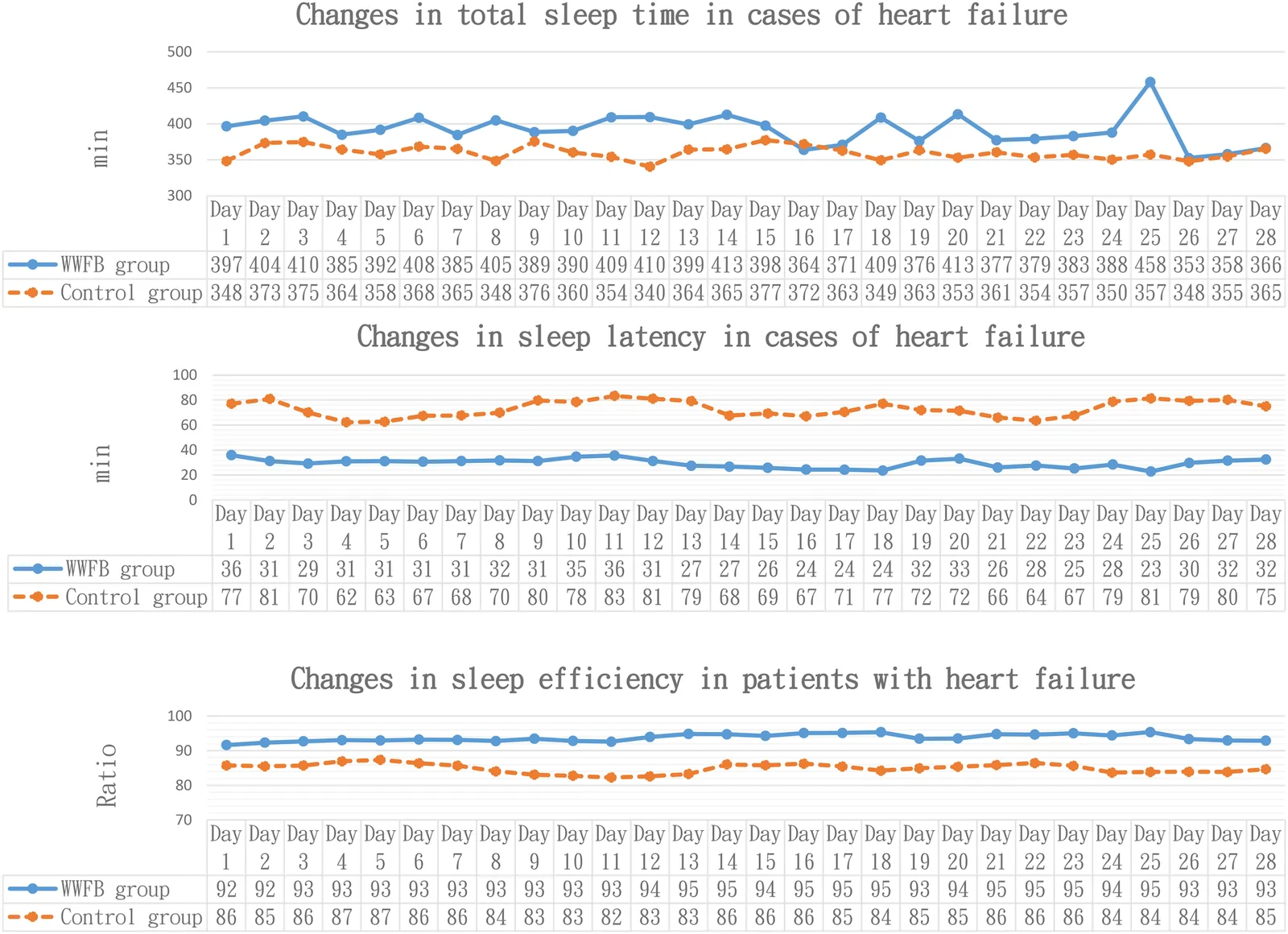
Changes in fatigue scale scores among patients with heart failure at pre-test, 4 weeks, and 8 weeks.

## 4 Discussion

To our knowledge, this is among the first randomized controlled studies to evaluate the effects of WWFBs on both sleep quality and fatigue in patients with HF. A total of 86 participants were recruited. Five participants withdrew because of insufficient time, six declined to complete the questionnaires, and one died because of disease progression. A total of 74 participants completed the 8-week follow-up period, resulting in a dropout rate of 14.0%. This dropout rate was higher than that reported by Raut et al. (24) in a shorter 5-day WWFB intervention, which may be related to the longer intervention duration in the present study. One participant in the experimental group died after four weeks because of worsening underlying cardiac condition (ejection fraction: 32%). This event was considered to be unrelated to the intervention based on clinical evaluation.

The results of this study showed that the subjective sleep quality of both groups improved significantly over time, with the experimental group demonstrating significantly greater improvement than the control group. Four weeks after the intervention, the experimental group showed a 1.27-point greater reduction in sleep quality score compared with the pre-intervention assessment, and eight weeks after the intervention, a 1.70-point greater reduction was observed. These findings are consistent with those of Guven et al. (25), who conducted a randomized controlled trial in community-dwelling older adults. In that study, the experimental group (*n* = 50) received a 20 min warm foot bath at 41–42 °C, with the water level 20 cm above the ankle, one hour before bedtime for six weeks. The results showed that the experimental group's sleep quality score improved significantly from 10.58 (SD = 3.91) before the intervention to 4.96 after the intervention. Similar to the present study, the PSQI scores gradually decreased over time, indicating a progressive improvement in subjective sleep quality. However, Guven et al. (25) reported a larger pre- and post-intervention difference (5.62 points), whereas the difference in this study was 2.1 points. This smaller improvement can be explained by several factors. First, Guven *et al*. required participants to have a PSQI score ≥ 5, indicating more severe baseline sleep disturbances than those in the present study. Second, the foot bath depth in the Guven *et al*. study was 20 cm above the ankle compared with 10 cm in this study. Third, the intervention duration was six weeks, longer than the four-week intervention used in the present study, which may have enhanced the therapeutic effect on sleep quality. Armat et al. (26) randomly assigned participants to a 40 °C foot bath group (*n* = 15), 37 °C foot bath group (*n* = 15), and control group (*n* = 15). The participants received a 10 min foot bath for two weeks. Both foot bath groups demonstrated significantly improved sleep quality compared to the control group. In the 40 °C group, the PSQI score decreased from 6.38 to 6.23 between weeks 2 and 4, while in the 37 °C group, the score decreased from 6.45 to 6.31. In contrast, the control group showed an increase from 8.42 to 8.48. These findings support the effectiveness of WWFBs at both temperatures in improving subjective sleep quality. Compared with that study, participants in the present study received four weeks of intervention, and the reduction in scores from week 4 to week 8 exceeded the reduction observed between weeks 2 and 4 in the study by Armat et al. (26), suggesting that a longer intervention duration may result in greater improvements in sleep quality. A related study by Ong (27) examined the effects of WWFBs on sleep quality in 20 older adult women. Participants received a 20 min foot bath at 40 °C twice weekly for two weeks before bedtime. The PSQI score decreased significantly from 8.75 before the intervention to 3.94 afterward. Although the scores in the present study were similar to those reported by Ong (27) at eight weeks after intervention (3.51), it is difficult to exclude a placebo effect in that study due to the small sample size, absence of a control group, and relatively low intervention frequency.

Regarding objective sleep parameters, including total sleep time, sleep latency, and sleep efficiency, no significant differences were observed between the groups in total sleep time. However, statistically significant between-group differences were observed for sleep efficiency on days 2, 9, 11, 12, 13, 18, 23, 24, and 25, whereas sleep latency differed significantly only on day 2. Kim et al. (28) conducted a study among older adults (*n* = 30) living in nursing homes, in which participants received 30 min foot baths daily for four weeks. The experimental group used 40 °C water, the placebo group used 36.5 °C warm water, and the control group received no intervention. Sleep status was measured using an activity recorder. WWFB interventions may improve subjective sleep quality and fatigue in patients with HF. However, objective sleep outcomes showed mixed results, with improvements observed mainly in sleep efficiency rather than total sleep duration or sleep latency. In the future, researchers should aim to include larger sample sizes and longer follow-up periods to confirm these findings. This study not only confirmed that WWFBs can effectively improve sleep quality in older adults but also indicated that the effect increased significantly during the second week, decreased slightly in the third week, and was sustained into the fourth week. In contrast, the present study did not show a statistically significant difference in total sleep time, but did demonstrate a statistically significant difference in sleep efficiency. This finding is similar to, and different from, the results reported by Kim et al. (28). This discrepancy may be explained by differences in the study populations, as participants in the present study were outpatients with HF, whereas those in Kim et al. (28) were older adults residing in nursing homes. Daily routines in nursing homes are often highly regulated, which may influence total sleep time.

Koike et al. (29) studied 13 hospitalized older adult patients with cognitive impairment who received 20 min foot baths at 42 °C, five days per week for two weeks, with sleep measured using an activity tracker. Their results showed no significant differences in total sleep time or sleep efficiency. These findings differ somewhat from those of the present study, possibly due to the smaller sample size and shorter intervention duration. Other studies have reported findings similar to those of Koike et al. (29), suggesting that WWFBs do not improve subjective or objective sleep quality. For example, Liao et al. (30) conducted a study of 15 older adults who received a 20 min foot bath at 41 °C at night, with sleep patterns and perceived sleep quality assessed; no significant differences were observed. Liao et al. (31) extended this work in 25 older adults with good and poor sleep quality using the same intervention and likewise reported no significant differences between groups. These findings differ from those of the present study, which may be because of differences in study design, intervention duration, and study population. The previous studies used crossover designs with single nightly foot bath interventions, which may have been insufficient to produce sustained physiological effects. In addition, the relatively small sample sizes may have limited statistical power and external validity. Taken together, these findings suggest that WWFB interventions may improve subjective sleep quality in patients with HF, although objective sleep outcomes remain inconsistent across studies.

WWFBs may influence heart rate and blood pressure by modulating autonomic nervous system activity, including decreased SNS activity and increased parasympathetic activity. Increased parasympathetic activity may contribute to vasodilation and a more relaxed physiological state, which may help alleviate fatigue (18). Peripheral warming may influence thermoregulation and promote sleep initiation through relaxation effects. In the present study, fatigue levels improved significantly over time in both groups, with a significantly greater improvement in the experimental group. Four weeks after the intervention, the experimental group demonstrated a 6.02-point reduction in fatigue compared to the pre-intervention levels, and at eight weeks, a 5.98-point reduction was observed. Although baseline fatigue scores differed between the groups, this randomized controlled trial employed GEE analysis, which adjusts post-intervention outcomes for baseline differences. Even with unequal pre-intervention values, GEE provides robust regression estimates by accounting for correlations across repeated measures. Therefore, baseline differences can be considered adequately controlled, allowing the post-intervention effect to be reliably evaluated. The findings indicate that patients with HF who received WWFB interventions experienced a sustained reduction in fatigue from baseline to week 4 and week 8, with greater improvement than the control group. These results suggest that WWFBs are effective in reducing fatigue in patients with HF. Consistent findings have been reported by Guler (32), who examined the effects of WWFBs on comfort, fatigue, and dialysis-related symptoms in older adult patients undergoing hemodialysis. In that study, the experimental group (*n* = 31) received 30 min foot baths at 40–43 °C during dialysis for four weeks and showed a significant reduction in fatigue scores from 7.17 at baseline to 3.48 post-intervention, whereas the control group (*n* = 27) showed no significant change. Similarly, Yang et al. (33) investigated the effects of WWFBs on fatigue in gynecological cancer patients undergoing chemotherapy. Participants in the experimental group soaked their feet in 41–42 °C water for 20 min in multiple phases over six months. Fatigue levels measured using the Mini-Fatigue Scale were significantly lower in the experimental group than in the control group, with sustained improvements throughout the study period. These findings align with those of the present study, demonstrating that WWFBs can significantly reduce fatigue over time. Choi and Song (34) also reported similar benefits in postpartum women, showing significantly greater reductions in fatigue scores among those who received foot bath interventions than among the controls.

Taken together, these findings suggest that both short- and long-term WWFB interventions at temperatures between 40 °C and 43 °C can effectively reduce fatigue, with fatigue levels decreasing progressively over time. Therefore, WWFBs may serve as an effective self-care strategy to alleviate fatigue in patients with HF.

This study employed convenience sampling, and due to limitations in manpower, time, resources, and funding, participants were recruited only from cardiology outpatient clinics at two medical centers in the Greater Taipei area. This limits the generalizability of the findings to a broader population of patients with HF. In addition, because the control group received routine care and the intervention required a detailed explanation, participants may have been aware of their group allocation, resulting in a lack of blinding and the potential influence of Hawthorne or placebo effects. Furthermore, the study was conducted from March to June (spring to summer), and seasonal factors may have affected participants' motivation and willingness to engage in WWFBs.

Future studies should consider multicenter designs across different geographic regions to increase sample size and representativeness. The present study involved a four-week intervention with an eight-week follow-up, demonstrating that the beneficial effects of WWFBs persisted beyond the intervention period. Future research should explore longer intervention durations, determine whether continued foot bath use is necessary to maintain the benefits, and examine the influence of different water temperatures and soaking durations. Additionally, with the growing popularity of herbal or essential oil foot baths, future studies should investigate their potential effects on sleep quality and fatigue using individualized intervention approaches.

In conclusion, our findings suggest that WWFBs may improve subjective sleep quality and reduce fatigue in patients with HF, although objective sleep outcomes showed mixed results. Improvements were observed mainly in sleep efficiency on selected study days, while limited effects were found for sleep latency and total sleep duration. These findings are partially consistent with those of previous studies, and these discrepancies may be influenced by differences in intervention duration, study population, and outcome measurements. WWFBs are non-invasive, easy to perform, and convenient for self-care practice. Given the high prevalence of fatigue and sleep disturbances in patients with HF, WWFBs may serve as a simple adjunctive intervention for symptom management in this population. Further studies with larger sample sizes and longer follow-up periods are needed to confirm these findings.

## Data Availability

The original contributions presented in the study are included in the article/Supplementary Material, further inquiries can be directed to the corresponding author/s.

## References

[1] Stahlman S Huizar-Garcia S Lipscomb J Frei C Oliver A. Implementation of a heart failure educational intervention for patients with recent admissions for acute decompensated heart failure. Front Cardiovasc Med. (2023):10:1133988. 10.3389/fcvm.2023.1133988 37215545 PMC10196446

[2] Zisapel N. Sleep and sleep disturbances: biological basis and clinical implications. Cell Mol Life Sci. (2007) 64:1174–86. 10.1007/s00018-007-6529-9 17364142 PMC11136060

[3] Chen Z Li M Yin C Fang Y Zhu Y Feng J. Effects of cardiac rehabilitation on elderly patients with chronic heart failure: a meta-analysis and systematic review. PLoS One. (2022) 17:e0273251. 10.1371/journal.pone.0273251 36006944 PMC9409506

[4] Vuckovic KM Ryan C Gomez Y Bierle R. Heart failure: an update for nurses. Nursing (Brux). (2023) 53:18–27. 10.1097/01.NURSE.0000927504.24034.9d 37212813

[5] Heidenreich PA Bozkurt B Aguilar D Allen LA Byun JJ Colvin MM. 2022 AHA/ACC/HFSA guideline for the management of heart failure: executive summary: a report of the American College of Cardiology/American Heart Association joint committee on clinical practice guidelines. Circulation. (2022) 145:e876–94. 10.1161/CIR.0000000000001062 35363500

[6] Spedale V Fabrizi D Rebora P Luciani M Alvaro R Vellone E. The association between self-reported sleep quality and self-care in adults with heart failure: a cross-sectional study. J Cardiovasc Nurs. (2023) 38:E98–109. 10.1097/JCN.0000000000000929 37027137

[7] Jorge-Samitier P Durante A Gea-Caballero V Antón-Solanas I Fernández-Rodrigo MT Juárez-Vela R. Sleep quality in patients with heart failure in the Spanish population: a cross-sectional study. Int J Environ Res Public Health. (2020) 17:7772. 10.3390/ijerph17217772 33114209 PMC7660645

[8] Lee KS Lennie TA Heo S Song EK Moser DK. Prognostic importance of sleep quality in patients with heart failure. Am J Crit Care. (2016) 25:516–25. 10.4037/ajcc2016219 27802953

[9] Polikandrioti M Kalafatakis F Koutelekos I Kokoularis D. Fatigue in heart failure outpatients: levels, associated factors, and the impact on quality of life. Arch Med Sci Atheroscler Dis. (2019) 4:e103–12. 10.5114/amsad.2019.85406 31211277 PMC6555087

[10] Hossein Pour AH Gholami M Saki M Birjandi M. The effect of inspiratory muscle training on fatigue and dyspnea in patients with heart failure: a randomized, controlled trial. Jpn J Nurs Sci. (2020) 17:e12290. 10.1111/jjns.12290 31429207

[11] Redeker NS Conley S Anderson G Cline J Andrews L Mohsenin V. Effects of cognitive behavioral therapy for insomnia on sleep, symptoms, stress, and autonomic function among patients with heart failure. Behav Sleep Med. (2020) 18:190–202. 10.1080/15402002.2018.1546709 30461315 PMC6529289

[12] Perez-Moreno AC Jhund PS Macdonald MR Petrie MC Cleland JGF Böhm M. Fatigue as a predictor of outcome in patients with heart failure: analysis of CORONA (controlled rosuvastatin multinational trial in heart failure). JACC Heart Fail. (2014) 2:187–97. 10.1016/j.jchf.2014.01.001 24720928

[13] Armbrister KA. Self-management: improving heart failure outcomes. Nurse Practitioner. (2008) 33:20–8. 10.1097/01.NPR.0000339206.36478.0f18941386

[14] Witte KK Clark AL. Why does chronic heart failure cause breathlessness and fatigue? Prog Cardiovasc Dis. (2007) 49:366–84. 10.1016/j.pcad.2006.10.003 17329182

[15] Chen DM Yu WC Hung HF Tsai JC Wu HY Chiou AF. The effects of baduanjin exercise on fatigue and quality of life in patients with heart failure: a randomized controlled trial. Eur J Cardiovasc Nurs. (2018) 17:456–66. 10.1177/1474515117744770 29189045

[16] Suna JM Mudge A Stewart I Marquart L O'Rourke P Scott A. The effect of a supervised exercise training programme on sleep quality in recently discharged heart failure patients. Eur J Cardiovasc Nurs. (2015) 14:198–205. 10.1177/1474515114522563 24491348

[17] Solmaz G. The effect of foot massage applied to patients with congestive heart failure on peripheral edema and sleep quality: a randomized controlled study. Holist Nurs Pract. (2023) 37:143–52. 10.1097/HNP.0000000000000581 37070839

[18] Ishikawa M. Effective combination of pupil light reflex and heart rate variability to assess foot bath effects on autonomic function in healthy adults. Biomed Phys Eng Express. (2020) 6:015034. 10.1088/2057-1976/ab6e1c 33438622

[19] Sawatari H Nishizaka MK Miyazono M Ando S-i Inoue S Takemoto M. Three nights leg thermal therapy could improve sleep quality in patients with chronic heart failure. Heart Vessels. (2018) 33:155–62. 10.1007/s00380-017-1047-7 28905211

[20] Buysse DJ Reynolds CF 3rd Monk TH Berman SR Kupfer DJ. The Pittsburgh sleep quality Index: a new instrument for psychiatric practice and research. Psychiatry Res. (1989) 28:193–213. 10.1016/0165-1781(89)90047-4 2748771

[21] Tsai P-S Wang S-Y Wang M-Y Su C-T Yang T-T Huang C-J. Psychometric evaluation of the Chinese version of the Pittsburgh sleep quality index (CPSQI) in primary insomnia and control subjects. Qual Life Res. (2005) 14:1943–52. 10.1007/s11136-005-4346-x 16155782

[22] Piper BF Dibble SL Dodd MJ Weiss MC Slaughter RE Paul SM. The revised piper fatigue scale: psychometric evaluation in women with breast cancer. Oncol Nurs Forum. (1998) 25:677–84. PMID: 9599351 9599351

[23] Chen L-H Li C-Y Shieh S-M Yin W-H Chiou A-F. Predictors of fatigue in patients with heart failure. J Clin Nurs. (2010) 19:1588–96. 10.1111/j.1365-2702.2010.03218.x 20579199

[24] Raut A Morade G More D Mune S Munjewar P Mute M. Effectiveness of warm water foot bath therapy on quality of sleep among elderly. Indian J Forensic Med Toxicol. (2021) 15:85–7. 10.37506/ijfmt.v15i2.14278

[25] Güven ŞD. Improving sleep quality in older adults: foot reflexology or warm footbath? A single-blind, 6-week intervention, two group, randomized comparative study. J Gerontol Nurs. (2022) 48:17–22. 10.3928/00989134-20220209-03 35201924

[26] Armat MR Mortazavi H Akbari H Ebrahimzadeh Z. The effect of footbath on the quality of sleep in older adults: a pilot study. Geriatr Nurs. (2021) 42:1178–83. 10.1016/j.gerinurse.2021.07.008 34419870

[27] Ong CJA. Effects of footbath in improving sleep quality among Filipino elderly. J Soc Health. (2018) 1:30–55.

[28] Kim H-J Lee Y Sohng K-Y. The effects of footbath on sleep among the older adults in nursing home: a quasi-experimental study. Complement Ther Med. (2016) 26:40–6. 10.1016/j.ctim.2016.02.005 27261980

[29] Koike Y Kondo H Kondo S Takagi M Kano Y. Effect of a steam foot spa on geriatric inpatients with cognitive impairment: a pilot study. Clin Interv Aging. (2013) 8:543–8. 10.2147/cia.s44005 23717038 PMC3663437

[30] Liao W-C Chiu M-J Landis CA. A warm footbath before bedtime and sleep in older Taiwanese with sleep disturbance. Res Nurs Health. (2008) 31:514–28. 10.1002/nur.20283 18459154 PMC2574895

[31] Liao W-C Wang L Kuo C-P Lo C Chiu M-J Ting H. Effect of a warm footbath before bedtime on body temperature and sleep in older adults with good and poor sleep: an experimental crossover trial. Int J Nurs Stud. (2013) 50:1607–16. 10.1016/j.ijnurstu.2013.04.006 23669188

[32] Güler S Şahan S Ülker T Sipahioğlu MH. The effect of footbath applied to patients receiving hemodialysis treatment on comfort, fatigue, and dialysis symptoms: a randomized controlled study. Ther Apher Dial. (2024) 28:23–33. 10.1111/1744-9987.14043 37779222

[33] Yang H-L Chen X-P Lee K-C Fang F-F Chao Y-F. The effects of warm-water footbath on relieving fatigue and insomnia of the gynecologic cancer patients on chemotherapy. Cancer Nurs. (2010) 33:454–60. 10.1097/ncc.0b013e3181d761c1 20562619

[34] Choi E Song E. Efficacy of a footbath for post-partum fatigue in South Korea: a quasi-experimental study. Jpn J Nurs Sci. (2017) 14:126–34. 10.1111/jjns.12143 27507794

